# *L*-Proline functionalized magnetic nanoparticles: A novel magnetically reusable nanocatalyst for one-pot synthesis of 2,4,6-triarylpyridines

**DOI:** 10.1038/s41598-018-35676-x

**Published:** 2018-11-23

**Authors:** Ali Maleki, Razieh Firouzi-Haji

**Affiliations:** Catalysts and Organic Synthesis Research Laboratory, Department of Chemistry, Iran University of Science and Technology, Tehran, 16846-13114 Iran

## Abstract

In this work, an efficient method for the immobilization of *L*-proline on magnetic nanoparticles was offered and evaluated as a recoverable magnetic nanocatalyst for synthesis of 2,4,6-triarylpyridines through one-pot three-component reaction of acetophenone, aryl aldehydes and ammonium acetate. This article is the first report of the catalytic application of *L*-proline functionalized magnetic nanoparticles in organic reactions as a magnetic nanocatalyst. This novel magnetic nanocatalyst proved to be effective and provided the products in high to excellent yield under solvent-free conditions. The structure of obtained nanoparticles was characterized by Fourier transform infrared spectrophotometry (FT-IR), field-emission scanning electron microscopy (FE-SEM), thermogravimetric analysis (TGA) and energy-dispersive X-ray spectroscopy (EDX). TGA result revealed that it is stable up to 200 °C for using as a catalyst in organic reactions. FE-SEM image of the synthesized nanocatalyst showed that it has nearly core-shell spherical shape and uniform size distribution with an average size about 80 nm. Moreover, the catalyst could be easily recovered by facile separation by magnetic forces and recycled for several times without significant loss of its catalytic activity. The benefits of this study are simplicity, nontoxicity, low cost, simple workup, and an environmentally benign nature.

## Introduction

In the recent years, organocatalysts have attracted increasing interest in organic synthesis particularly from the green chemistry points of view^1–4^. Organocatalysts are metal-free small organic molecules that are able to function as efficient and selective catalysts for a wide range of organic reactions. Among them, L-proline and its derivatives have considered as powerful organocatalysts^5^. L-Proline has been successfully applied in many reactions, such as Robinson annulations, aldol reactions, Mannich reactions, Michael reactions, direct electrophilic α-aminations, Diels–Alder reactions, Baylis–Hillman reactions, aza-Morita-Baylis–Hillman reactions, *α*-selenenylation, oxidation, chlorination, and others^6–11^. Recently, immobilization and recycling of L-proline have received considerable concerns and there are several types of supports for the immobilizations of proline and its derivatives such as polymer, silica, ionic liquid, cyclodextrin, and magnetite^12–16^. Furthermore, magnetic nanoparticles (MNPs) have recently considered as a new type of catalyst support for organocatalysts due to their price, high dispersion, good stability, easy synthesis and functionalization method, high surface area and facile separation by using external magnetic fields^17–49^.

Multicomponent reactions (MCRs) as an important organic synthesis strategy are one-pot process in which three or more accessible substrate react to produce a more complex molecule that essentially includes most or all atoms of the starting materials^50^. Pyridines are nitrogen-containing heterocyclic compounds which have received significant attention because of their various medicinal, biological and pharmaceutical activities such as hypoglycemic activity, hypolipidemic activity, fungicidal activity, antimicrobial agent, dopamine transporter inhibitors and anti-inflammatory agents^51–53^. In addition, pyridines are used in supramolecular chemistry due to their π-stacking ability^54^. Among all of the pyridine derivatives, 2,4,6-triarylpyridines have received much interest by organic chemists due to their importance in medicinal chemistry. Because of significant features of these heterocyclic scaffolds, many efficient protocols were developed to more efficient synthesis of 2,4,6-triarylpyridines, for example solid-phase synthesis^55^, one-pot synthesis under microwave irradiation^56^, and solvent-free reaction between acetophenones, benzaldehydes, and ammonium acetate in the presence of various catalyst such as nanoparticles^57^, heteropolyacid^58^, HClO_4_–SiO_2_^59^, and ionic liquid^60^. However, most of these methods suffer from drawbacks such as long reaction time, harsh reaction conditions, the use of volatile organic solvents, low yields, high catalyst loading, thermal conditions and expensive or difficult procedures of catalyst preparation. Therefore, design and development of mild and efficient methods with more environmentally-friendly catalysts is in of prime importance.

**Figure 1:**
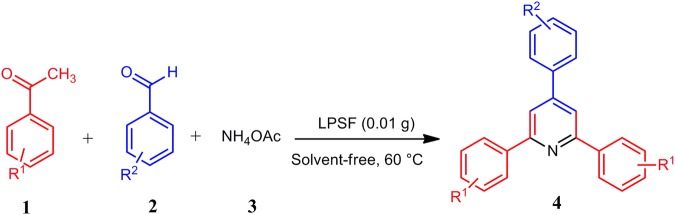
Synthesis of 2,4,6-triarylpyridines **4a–k** in the presence of LPSF nanocatalyst.

In continuation of our research on the introduction of recoverable catalysts in organic synthesis^61–63^, herein, we report a convenient and facile one pot synthesis of 2,4,6- triarylpyridines from acetophenones **1** (2 mmol), aromatic aldehydes **2** (1 mmol) and NH_4_OAc **3** (1.5 mmol) in the presence of Fe_3_O_4_\SiO_2_\propyltriethoxysilane\L-proline nanoparticles (LPSF) nanoparticle, as heterogeneous catalyst at 60 °C under solvent-free conditions (Fig. 1). To the best of our knowledge, this synthesized nanocatalyst was synthesized and applied as a novel, efficient and eco-friendly nanocatalyst in chemical reactions, especially in the synthesis of 2,4,6- triarylpyridines **4a–k**.

## Results and Discussion

**Figure 2:**
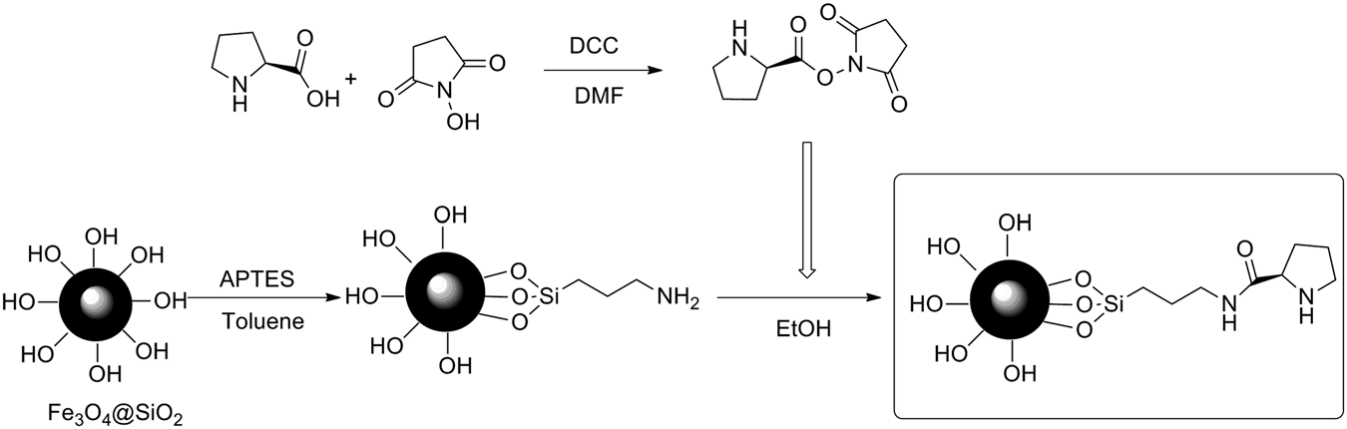
Preparation of LPSF magnetic nanocatalyst.

In this work, we have synthesized a novel nanomagnetic organocatalyst Fe_3_O_4_\SiO_2_\propyltriethoxysilane\L-proline (LPSF) and applied for the synthesis of 2,4,6-triarylpyridines. As can be seen in Fig. 2, LPSF nanocatalyst was prepared after several steps. At first, L*-*proline *N-*hydroxysuccinimide ester was prepared from L-proline and N-hydroxy succinimide (NHS) in the presence of *N,N’*-dicyclohexylcarbodiimide (DCC). After that, the synthesized Fe_3_O_4_\SiO_2_ was treated by (3-aminopropyl)triethoxysilane (APTES) to synthesize Fe_3_O_4_\SiO_2_\3-aminopropyltriethoxysilane. Finally, the synthesize Fe_3_O_4_\SiO_2_\3-aminopropyltriethoxysilane was treated by NHS-L-proline to synthesize the aimed LPSF magnetic nanocatalyst. Then, the characterizations of the prepared nanocomposite were investigated by several analyses methods which will be discussed further.

### Characterization of the prepared Fe_3_O_4_\SiO_2_\propyltriethoxysilane\L-proline (LPSF)

#### FT-IR spectra

**Figure 3:**
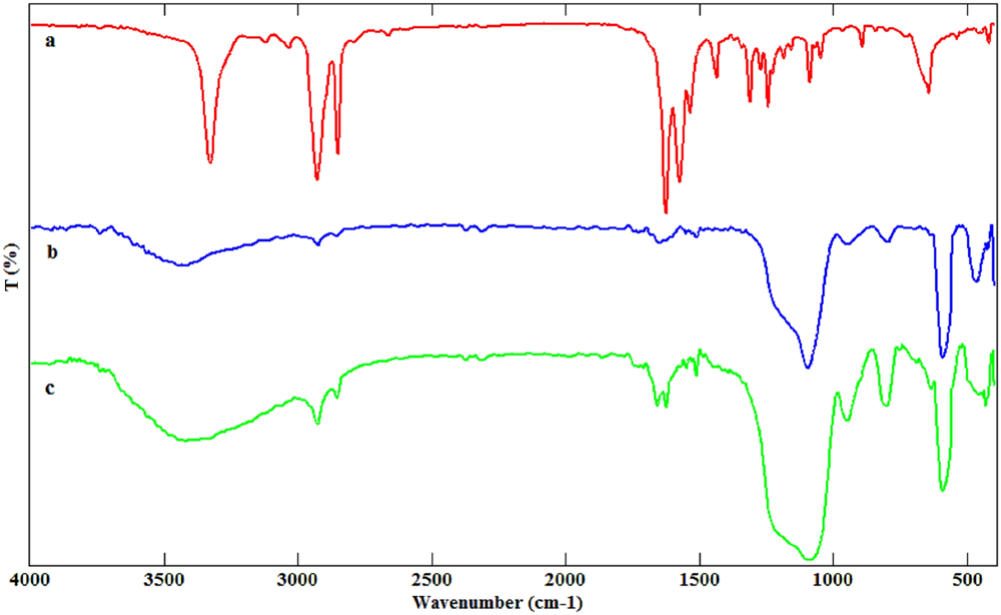
FT-IR spectra of: (**a**) NHS-L-proline, (**b**) Fe_3_O_4_\SiO_2_\3-aminopropyltriethoxysilane, (**c**) LPSF magnetic nanocatalyst.

As can be seen in Fig. 3, the FT-IR spectrum of the LPSF magnetic nanocatalyst can verify the preparation of the expected product. The bending vibration band at 585 cm^−1^ is indicated Fe–O vibration. In addition, the sharp bands appearing at 1084 and 1120 cm^−1^ are attributed to Si–O–Si asymmetric stretching vibration confirmatory to the SiO_2_ formation. The asymmetric and symmetric aromatic C–H stretching vibrations are appeared at 2920 and 2852 cm^−1^. Furthermore, the asymmetric stretching vibrations of O–H and N–H groups observed at 3401 cm^−1^. Furthermore, we have characterized the recycled LPSF magnetic nanocatalyst. As shown in Fig. S1, there was no considerable deformation or leaching after seven times reusing.

#### FE-SEM images

**Figure 4:**
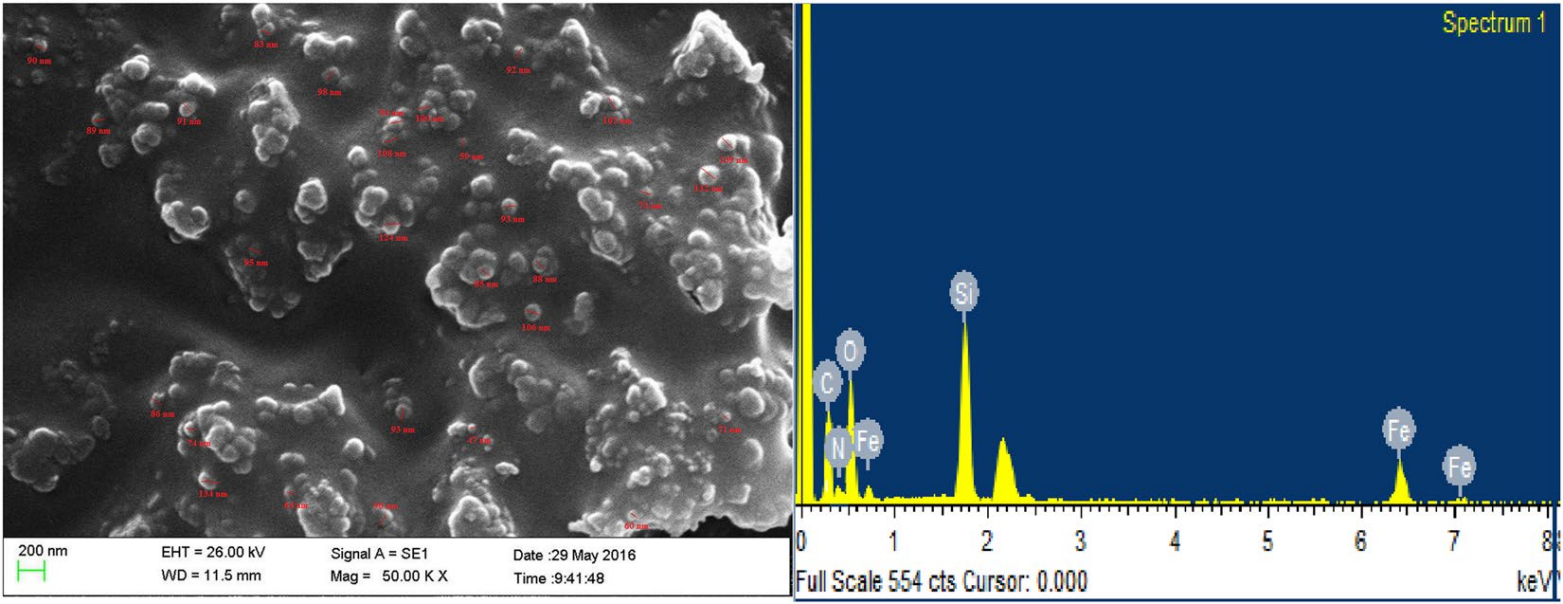
(**a**) FE**-**SEM image and (**b**) EDX analysis of LPSF magnetic nanocatalyst.

Field-emission scanning electron microscopy (FE-SEM) images are used to investigate the surface structure of the nanocomposite. As it is seen in Fig. 4a, FE-SEM images show that the LPSF nanocatalyst has nearly spherical shape and uniform size distribution with an average size of 80 ± 40 nm.

#### EDX analysis

The result of the EDX analysis of LPSF magnetic nanocatalyst is illustrated in Fig. 4b. It confirms the presence of C, Fe, N, Si and O atoms elements in the nanocatalyst.

#### Thermal analysis

**Figure 5:**
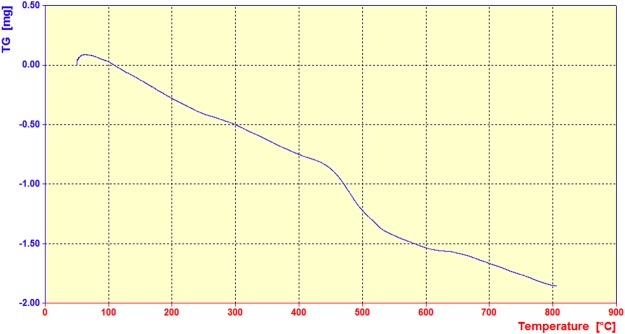
TG curve of LPSF nanocatalyst.

As can be seen in Fig. 5, the thermal behaviour of the prepared nanocomposite was evaluated by thermogravimetric analysis (TGA) over the temperature range of 20–800 °C at air atmosphere. The first weight loss between 0–100 °C was due to evaporation of adsorbed water in the sample. After that, the weight loss from 200 to 600 is related to the destruction of the organic compounds.

### Catalytic application of Fe_3_O_4_\SiO_2_\propyltriethoxysilane\L-proline (LPSF) in the synthesis of 2,4,6-triarulpyridines

The catalytic activity of the synthesized magnetic nanocatalyst was investigated in a one-pot three-component synthesis of 2,4,6-triarylpyridine derivatives. Initially, to optimize the reaction conditions, we evaluated the reaction of acetophenone, 4-chloro-benzaldehyde and ammonium acetate in the presence of different catalytic amounts of LPSF magnetic nanocatalyst at 60 °C under solvent-free conditions, as a model reaction to yield **4b**. It was observed that 0.01 g of catalyst was enough to catalyze the reaction to produce high yields of products (Table S1 in Supplementary Information file). To study of the solvent effect and comparing the efficiency of ethanol, the model reaction was performed in several solvents with different polarities in the presence of LPSF magnetic nanocatalyst. As can be seen in Table S1 (Entries 6–9), the efficiency and the yield of the model reaction under solvent-free conditions were higher than those obtained in other solvents.

In addition, a comparison was done between the present work and others earlier reports for the synthesis of **4b**. The results summarized in Table S2 in Supplementary Information file clearly demonstrate the superiority of the present work in saving energy, high yields of the products and the reusability of the nanocatalyst.

Finally, in order to examine the generality of this nanocatalyst for the synthesis of 2,4,6-triarylpyridine derivatives, a number of aromatic aldehydes and acetophenones with electron-withdrawing and electron-releasing substitutions, were employed and a variety of products were synthesized under the optimized conditions the results are summarized in Table 1.

**Table 1:** Synthesis of 2,4,6-triarylpyridines 4a–k by using LPSF magnetic nanocatalyst.

Entry	R^1^	R^2^	Product	Time (min)	Yield^a^ (%)	Mp (°C)	
						Observed	Literature
1	H	H	**4a**	60	88	134–137	135–137^64^
2	H	4-Cl	**4b**	60	94	121–123	123–124^65^
3	H	4-NO_2_	**4c**	60	91	193–194	196–198^64^
4	H	4-OH	**4d**	60	88	199–200	196–198^65^
5	H	4-Me	**4e**	60	83	117–120	119–120^56^
6	H	4-Br	**4f**	60	90	162–163	165–166^65^
7	H	4-OMe	**4g**	60	83	98–100	97–98^66^
8	4-Cl	H	**4h**	60	84	180–181	175–178^56^
9	4-Cl	4-OMe	**4i**	60	88	188–189	190–191^67^
10	4-Me	4-Cl	**4j**	60	89	162–164	159–160^64^
11	H	furan-2-carbaldehyde	**4k**	60	75	110–112	112–115^68^

^a^Isolated yield.

### Mechanistic evaluation

**Figure 6:**
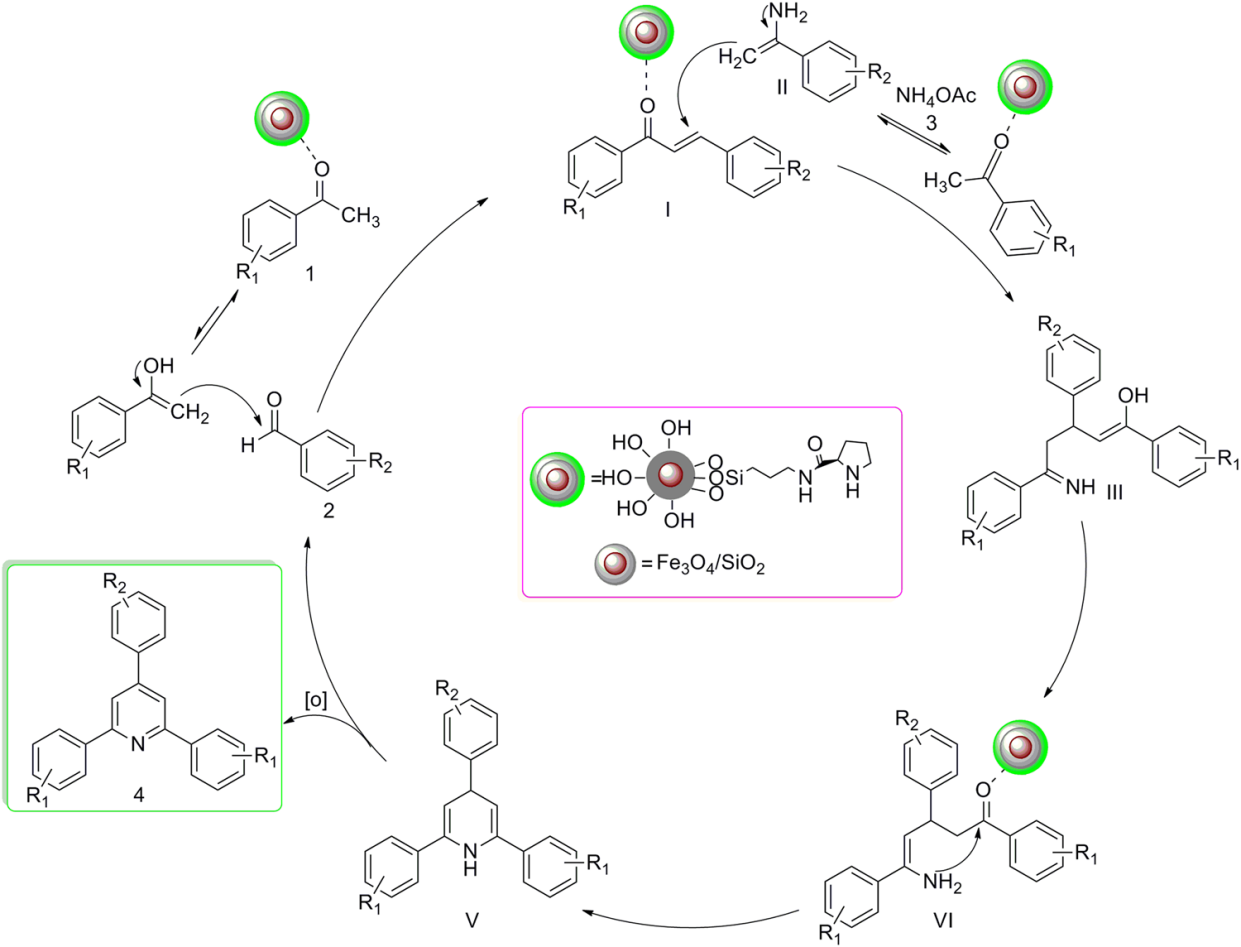
Plausible mechanism for the formation of 2,4,6-triarylpyridines (**4a–k**).

The plausible mechanism for the formation of 2,4,6-triarylpyridines is shown in Fig. 6. The first step is formation of an intermediate **I**, formed *via* Aldol condensation of an aromatic aldehyde **2** and acetophenones **1** in the presence of LPSF magnetic nanocatalyst. After that, enamine **II** is formed via condensation of the other molecule of acetophenones and ammonium acetate **III**. In continuation of the reaction, a Michael addition is occurred between intermediate **I** and enamine **II** to afford intermediate **III**. Then, cyclization of intermediate **IV** leads to produce dihydropyridine **V**. Finally, oxidation takes place in the presence of LPSF magnetic nanocatalyst to afford final product **4**.

### Recyclability of LPSF magnetic nanocatalyst

In order to investigate the possibility of several recycling runs for LPSF magnetic nanocatalyst, the solid catalyst was separated from the reaction mixture by using an external magnet. It was washed two times with ethanol and water, dried and reused in subsequent reactions. The catalyst can be reused seven times without any significant decrease in yield of the products (Fig. S1). Finally, as can be seen in Figs S2 and S3, we have characterized the recycled nanocatalyst by FT-IR spectroscopy and FE-SEM image which showed suitable retention of its structure and morphology.

## Experimental

### General

All the solvents, chemicals and reagents were purchased from Merck, Sigma and Aldrich. Melting points were measured on an Electro thermal 9100 apparatus and are uncorrected. Fourier transforms infrared spectroscopy (FT-IR) spectra were recorded on a Shimadzu IR-470 spectrometer by the method of KBr pellet. ^1^H and ^13^C NMR spectra were recorded on a Bruker DRX-300 Avance spectrometer at 500 and 125 MHz, respectively. Field-emission scanning electron micrograph (FE-SEM) images were taken with Sigma-Zeiss microscope with attached camera. Elemental analysis of the nanocatalyst was carried out by energy-dispersive X-ray (EDX) analysis recorded Numerix DXP-X10P. Thermal analysis was taken by Bahr-STA 504 instrument under argon atmosphere.

### Preparation of Fe_3_O_4_\SiO_2_\propyltriethoxysilane\L-proline nanoparticles

#### Preparation of compound L-proline N-hydroxysuccinimide ester

In the first step, 0.24 g L-proline and 0.45 g N-hydroxy succinimide (NHS) were mixed in 35 mL of DMF and vigorously stirred under 35 °C until the two components were completely dissolved. Then, 0.75 g *N,N’*-dicyclohexylcarbodiimide (DCC) was added gradually and the reaction mixture were stirred for 24 h. After that, white precipitate L*-*proline N*-*hydroxysuccinimide ester (NHS-L-proline) was separated after drying solvent by rotary and washed with diethyl ether.

#### Preparation of Fe_3_O_4_ nanoparticles

The Fe_3_O_4_ nanoparticles were synthesized *via* the coprecipitation of FeCl_3_·6H_2_O and FeCl_2_·4H_2_O at a molar ratio of 2: 1 in the presence of ammonia. Typically, 2.82 g of FeCl_3_·6H_2_O and 1.72 g of FeCl_2_·4H_2_O were mixed in 80 mL of distilled water and vigorously stirred at 80 °C with a mechanical stirrer. After the temperature had reached 80 °C, 10 mL ammonia was added drop wise to the mixture. The mixture was then stirred for another 40 min and then cooled to room temperature. The black precipitate was collected using an external magnet and washed several times with ethanol and distilled water. The black product was dried at 80 °C in an oven.

#### Preparation of Fe_3_O_4_\SiO_2_ nanoparticles

Initially, 45 mg of Fe_3_O_4_ nanoparticles were dispersed in 16 mL of deionized water by using an ultrasonic water bath, after that 2 mL of aqueous ammonia solution (25 wt%) and 80 mL of ethanol were added to reaction mixture. Next, 0.8 mL of tetraethyl orthosilicate (TEOS) was added drop wise into the Fe_3_O_4_ nanoparticle solution under vigorous stirring at room temperature. The mixture was then stirred for 24 h at room temperature. The products were separated by an external magnet and washed several times with distilled water. The final product was collected and dried at 50 °C.

#### Preparation of Fe_3_O_4_\SiO_2_\3-aminopropyltriethoxysilane nanoparticles

At first, 1 g obtained Fe_3_O_4_@SiO_2_ nanocomposite was added in 5 mL of toluene and ultrasonicated for 10 min. Then, 2 mL (3-aminopropyl)triethoxysilane (APTES) was added to this solution and refluxed for 18 h. The obtained amino-substituted nanocomposites were separated by an external magnet and washed two times with toluene and washed. After that, collected nanocomposite was extracted and washed in toluene using a Soxhlet apparatus in toluene for removing unreacted starting materials. The precipitation was dried at 60 °C for 12 h.

#### Preparation of Fe_3_O_4_\SiO_2_\propyltriethoxysilane\L-proline nanoparticles (LPSF)

The obtained Fe_3_O_4_@SiO_2_@OSi(CH_2_)_3_NH_2_ (1 g) was dispersed in with 15 mL ethanol. Then NHS-L-proline (1 g) was added into the above solution under vigorous stirring. The obtained mixture was stirred for 6 h at room temperature. After completion of the reaction, the products were separated by an external magnet and washed several times with ethanol. The precipitation was dried at 50 °C for 12 h.

#### General procedure for preparing 2,4,6-triarylpyridines

A mixture of acetophenones (2 mmol), aromatic aldehyde (1.0 mmol), ammonium acetate (1.5 mmol) and 0.01 g LPSF nanocatalyst was stirred at 60 °C under solvent-free conditions for an appropriate time. The completion of the reaction was monitored by thin layer chromatography (TLC). After completion of the reaction, hot ethanol was added to the mixture added to the mixture and the catalyst was separated easily by an external magnet. The pure products were obtained from the reaction mixture by recrystallization from hot EtOH and no more purification was required. All the product were known compounds which were identified by characterization of their melting point with those authentic literature samples and also in some cases their ^1^H and ^13^C NMR spectral data.

### Selected Spectral data

4-(4-Nitrophenyl)-2,6-diphenylpyridine (**4c**): White solid; ^1^H NMR (500 MHz, CDCl_3_): δ_H_ (ppm) = 7.47–7.57 (8 H, m, H-Ar), 7.70 (2 H, d, *J* = 8.0 Hz, H-Ar), 7.86 (2 H, s, H-Ar), 8.23 (4 H, d, *J* = 6.3 Hz, H-Ar); ^13^C NMR (125 MHz, CDCl_3_): δ_C_ (ppm) = 117.2, 127.6, 128.8, 129.2, 129.6, 129.8, 135.6, 137.9, 139.8, 149.4, 158.1.

## Conclusions

In summary, LPSF magnetic nanocatalyst was prepared and used as a novel, green, magnetically recyclable, environmentally-friendly and efficient composite nanocatalyst for the synthesis of chemically and biologically important 2,4,6-triarylpyridines by a simple, clean, eco-friendly and inexpensive method. The novel magnetic nanocatalyst can be easily separated by an external magnet and recycled for several times without any significant loss of activity. We used FT-IR, EDX, TGA and FE-SEM to confirm that the nanocomposite was formed, and ^1^H and ^13^C NMR analyses were performed for the confirmation of the synthesized products. TGA result revealed that it is stable up to 200 °C for using as a catalyst in organic reactions. FE-SEM image of the synthesized nanocatalyst showed that it has nearly core-shell spherical shape and uniform size distribution with an average size about 80 nm. This study is the first report on design, synthesis, functionalization and characterization of the novel magnetic nanocomposite and also performance as a heterogeneous catalyst in organic reactions.

## Electronic supplementary material

Supplementary Information
