# Supplementary material for: L-Proline functionalized magnetic nanoparticles: A novel magnetically reusable nanocatalyst for one-pot synthesis of 2,4,6-triarylpyridines
Source: Sci Rep. 2018 Nov 23;8:17303. doi: 10.1038/s41598-018-35676-x (PMC6251865; doi:10.1038/s41598-018-35676-x)
Supplement: Supplementary file 1 — Supplementary Information [file 41598_2018_35676_MOESM1_ESM.doc]

***L*-Proline functionalized magnetic nanoparticles: A novel magnetically reusable nanocatalyst for one-pot synthesis of 2,4,6-triarylpyridines**

Ali Maleki* and Razieh Firouzi-Haji

*Catalysts and Organic Synthesis Research Laboratory, Department of Chemistry, Iran University of Science and Technology, Tehran 16846-13114, Iran*

**Corresponding author E-mail:* [*maleki@iust.ac.ir*](mailto:maleki@iust.ac.ir)*; Fax: +98-21-73021584; Tel: +98-21-77240540-50*

| **Table of contents** |
| --- |
| **Subject Page** |
| Table S1. Optimization of reaction conditions in the synthesis of the product **4b**…………….……………S2  Table S2. Comparison of some catalysts effects with LPSF magnetic nanocatalyst on the model reaction..S3  Figure S1. Recycling diagram of LPSF magnetic nanocatalyst in the synthesis of **4b**………………...…...S4  Figure S2. FT-IR spectra of (a) LPSF, (b) Recycled LPSF…………………………………………………S5  Figure S3. FE-SEM image of recycled LPSF magnetic nanocatalyst………………………………………S6  Figure S4. ^1^H NMR spectrum of the product **4b** ..……………………………………..………...………...S7 |
| Figure S5. ^13^C NMR spectrum of the product **4b** ………………………………………...….…..…...…...S8  Figure S6. ^1^H NMR spectrum of NHS-L-proline. …………………………………………….…..…...…...S9 |

**Table S1.** Optimization of reaction conditions in the synthesis of the product **4b**.

| Entry | Catalyst (g) | Solvent | Time (min) | Yield^a^ (%) |
| --- | --- | --- | --- | --- |
| 1 | - | - | 60 | Trace |
| 2 | 0.002 | - | 60 | 80 |
| 3 | 0.006 | - | 60 | 84 |
| 4 | 0.008 | - | 60 | 90 |
| 5 | 0.01 | - | 60 | 94 |
| 6 | 0.02 | - | 60 | 94 |
| 7 | 0.01 | H_2_O | 60 | 55 |
| 8 | 0.01 | CH_3_CN | 60 | 60 |
| 9 | 0.01 | EtOH | 60 | 87 |

^a^ Isolated yield.

**Table S2.** Comparison of some catalysts effects with LPSF magnetic nanocatalyst on the model reaction.

| Entry | Catalyst | Catalyst amount (g) | Temp. (°C) | Time (min) | Yield^a^ (%) |
| --- | --- | --- | --- | --- | --- |
| 1 | CoCl_2_.6H_2_O | 0.12 | 110 | 240 | 91^33^ |
| 2 | Nano-titania-supported sulfonic acid | 0.009 | 110 | 100 | 95^34^ |
| 3 | - | - | 120 | 130 | 81^35^ |
| 3 | Fe_3_O_4_/SiO_2_ | 0.01 | 60 | 60 | 85 |
| 4 | Fe_3_O_4_\SiO_2_\3-amino propyltriethoxysilane | 0.01 | 60 | 60 | 89 |
| 5 | LPSF | 0.01 | 60 | 60 | 94 |

^a^ Isolated yield


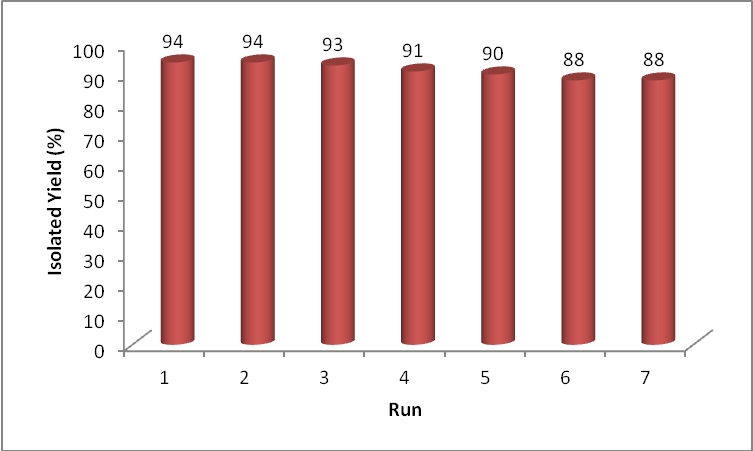


**Figure S1.** Recycling diagram of LPSF magnetic nanocatalyst in the synthesis of **4b**.


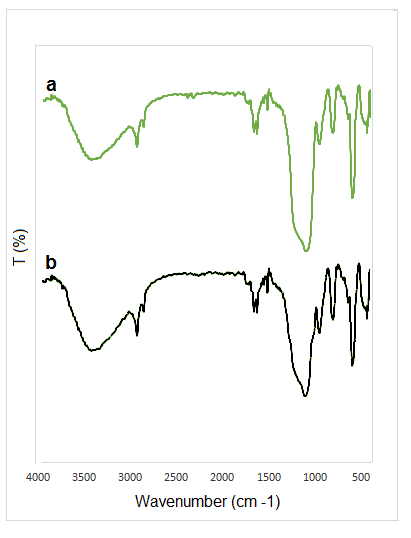


**Figure S2.** FT-IR spectra of (a) LPSF, (b) the recycled LPSF.

**
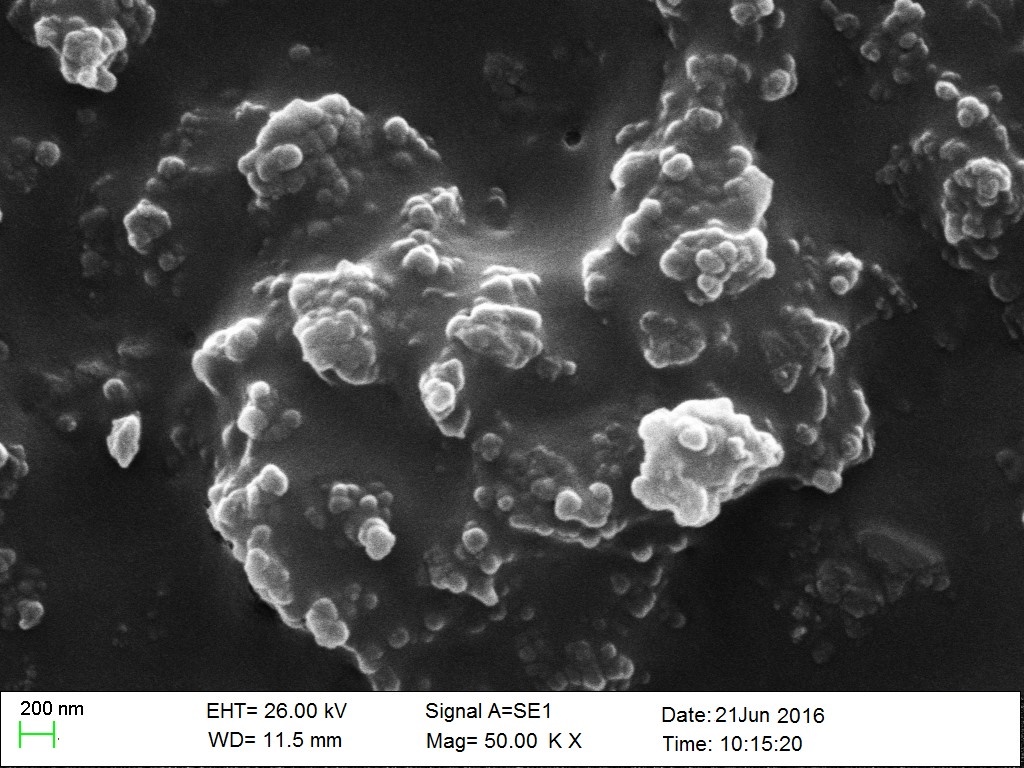
**

**Figure S3.** FE**-**SEM image of the recycled LPSF magnetic nanocatalyst.


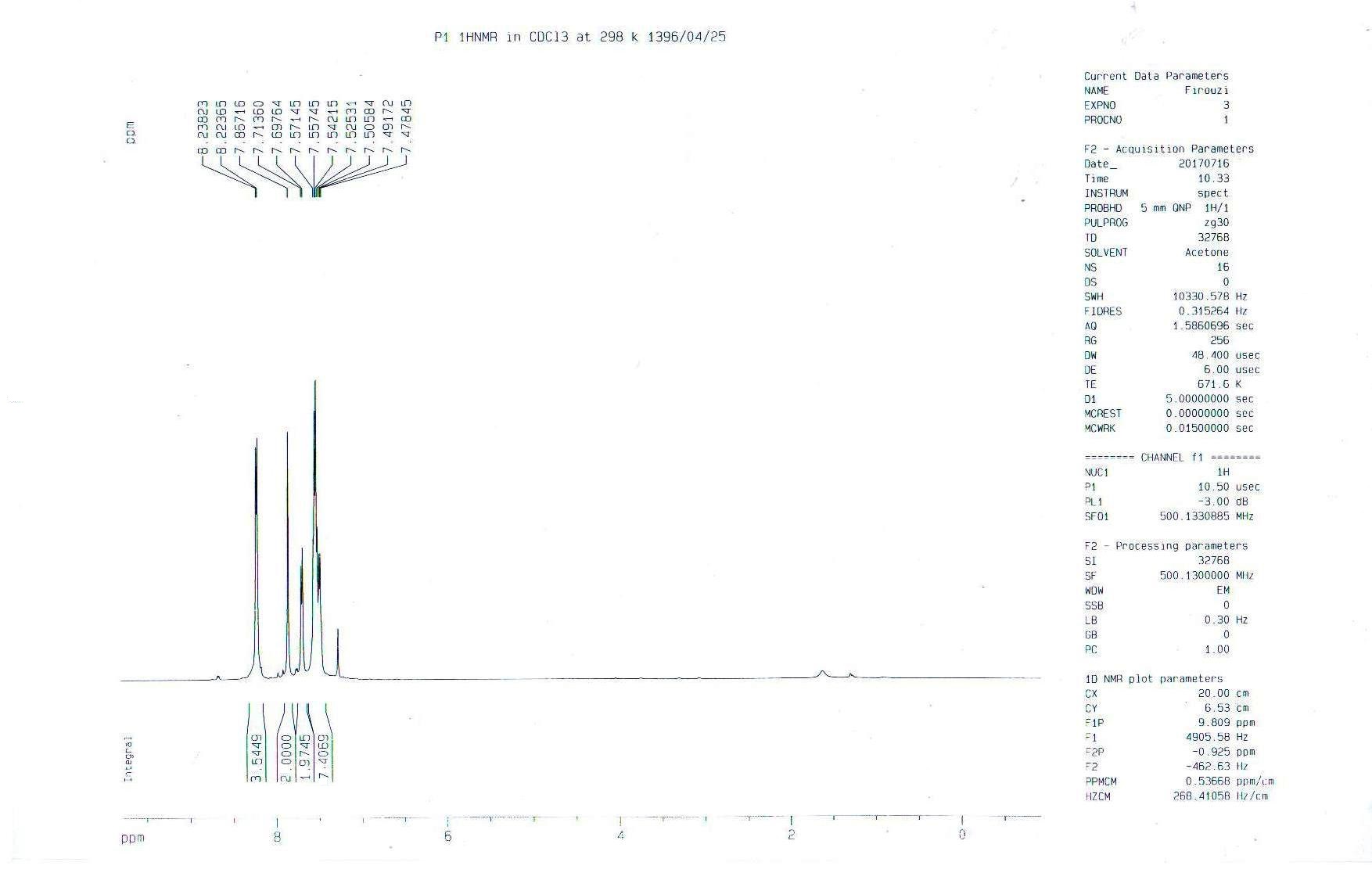


**Figure S4.** ^1^H NMR spectrum of the product **4b**.


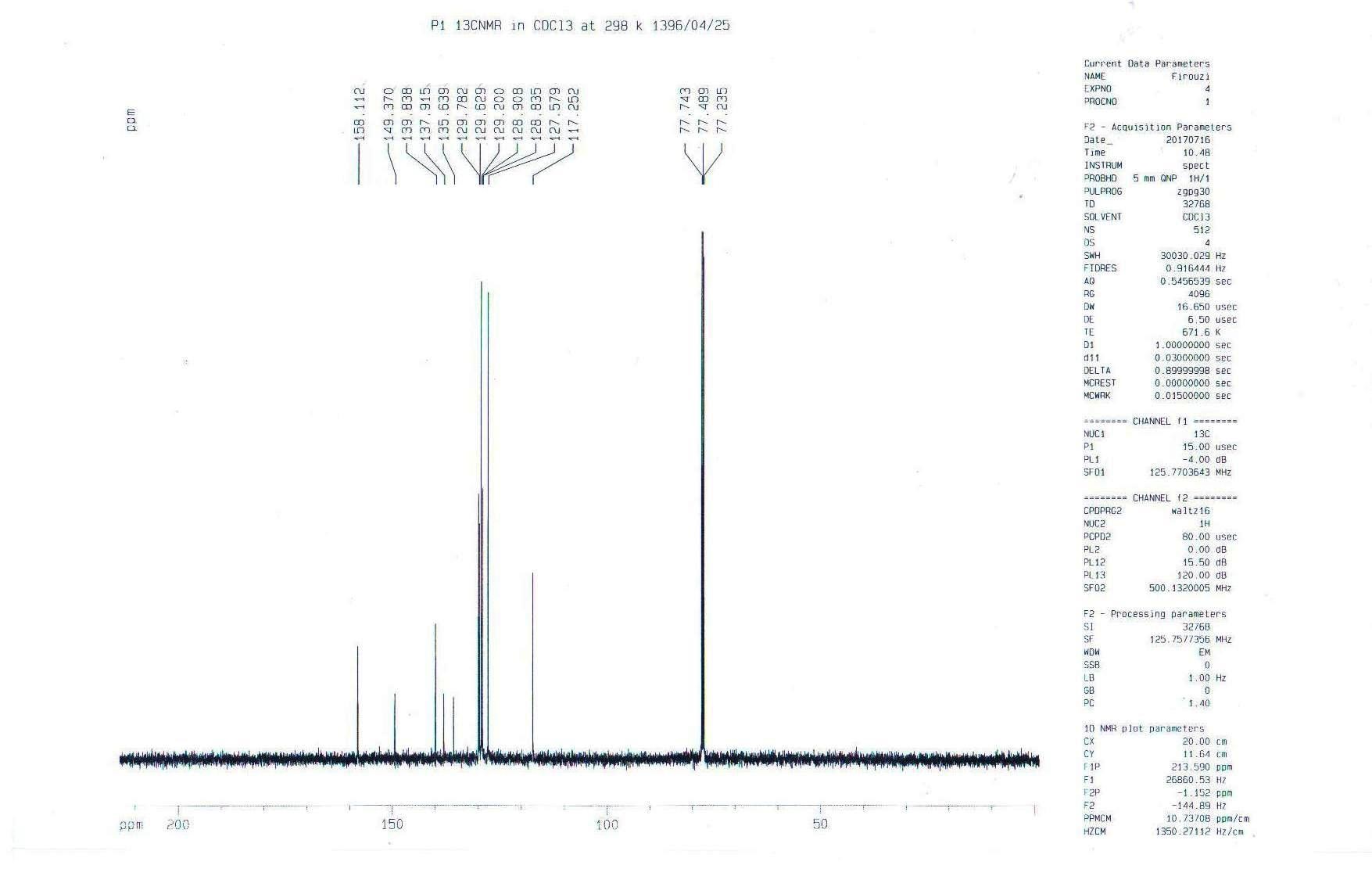


**Figure S5.** ^13^C NMR spectrum of the product **4b**.


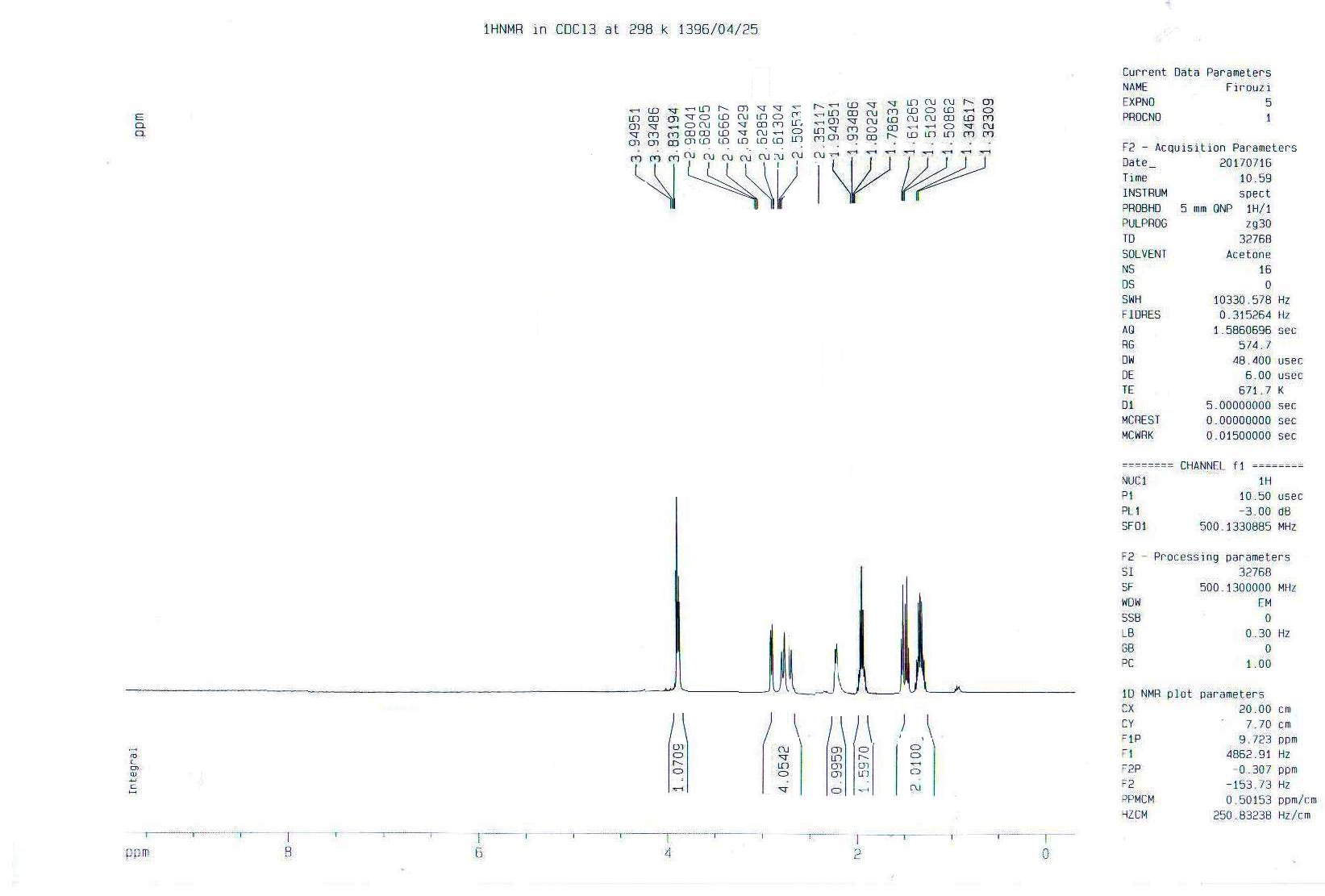


**Figure S6.** ^1^H NMR spectrum of NHS-L-proline.
